# Supplementary material for: Hybrid Bis-Histidine Phenanthroline-Based Ligands to Lessen Aβ-Bound Cu ROS Production: An Illustration of Cu(I) Significance
Source: Molecules. 2021 Dec 16;26(24):7630. doi: 10.3390/molecules26247630 (PMC8707446; doi:10.3390/molecules26247630)
Supplement: Supplementary file 1 [file molecules-26-07630-s001.zip › molecules-1508821-supplementary.pdf]

molecules

MDPI

Article

Supporting Information for

Hybrid Bis-Histidine Phenanthroline-Based Ligands to Lessen Aβ-Bound Cu ROS Production: An Illustration of Cu(I) Significance

Marielle Drommi[1], Clément Rulmont[1], Charlène Esmieu[1] and Christelle Hureau[1,*]

[1] CNRS, LCC (Laboratoire de Chimie de Coordination), 205 route de Narbonne, 31077 Toulouse cedex 4, France
* Correspondence: christelle.hureau@lcc-toulouse.fr

Citation: Drommi, M.; Rulmont, C.; Esmieu, C.; Hureau, C. Hybrid Bis-Histidine Phenanthroline-Based Ligands to Lessen Aβ-Bound Cu Ros Production: An Illustration of Cu(I) Significance. *Molecules* **2021**, *26*, 7630. https://doi.org/10.3390/molecules26247630

Academic Editor: Farkas Etelka

Received: 29 November 2021
Accepted: 10 December 2021
Published: 16 December 2021

Publisher's Note: MDPI stays neutral with regard to jurisdictional claims in published maps and institutional affiliations.

Copyright: © 2021 by the authors. Licensee MDPI, Basel, Switzerland. This article is an open access article distributed under the terms and conditions of the Creative Commons Attribution (CC BY) license (http://creativecommons.org/licenses/by/4.0/).

1. Synthesis

Products were characterized by:

-^{1}H, ^{13}C, COSY, HMQC and HMBC nuclear magnetic resonance (NMR) recorded on a Bruker Avance 300, Avance 400 or AvanceIII 400.

-DCI NH_3 mass spectrometry (MS) recorded on a DSQ II (Thermo Fisher Scientific) or ESI-MS recorded on a Q-TOF Premier (Waters).

-UV-visible spectroscopy recorded on a Hewlett Packard Agilent 8453 spectrophotometer

-Infrared spectroscopy (IR) recorded on a Perkin Elmer FT-IR Frontier spectrophotometer, with an ATR crystal (GladiATR, Pike technologies).

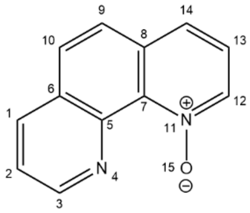

1,10-phenanthroline-1-oxide. In a 250 mL flask, 10 g (55 mmol) of 1,10-phenanthroline were dissolved in the minimal amount of glacial acetic acid (10 mL) at 70-75 °C. Then 30 mL of 30% hydrogen peroxide were added dropwise to the reaction mixture and the mixture was maintained at 70-75 °C. The disappearance of 1,10-phenanthroline and the appearance of 1,10-phenanthroline were followed by thin layer chromatography (TLC) with dichloromethane/methanol (90/10 v/v) as eluent, and by 1H NMR. Additional 30 mL of 30% hydrogen peroxide were added dropwise to the mixture every 3 hours until the complete disappearance of 1,10-phenanthroline was achieved. After cooling, the mixture was basified to pH ≈ 10-12 with a concentrated potassium hydroxide solution. The mixture was extracted 10 times with 100 mL fractions of dichloromethane. The combined extracts were dried over magnesium sulfate, filtrated and evaporated to give a yellow solid (7.6 g, 71% yield). The product was engaged in the next step without further purification. **^{1}H NMR** (300 MHz, $CDCl_3$) δ 9.31 (dd, $^{3}J_{12\text{-}13}$ = 4.4 Hz, $^{4}J_{12\text{-}14}$ = 1.9 Hz, 1H_{12}), 8.75 (dd, $^{3}J_{2\text{-}3}$ = 6.3 Hz, $^{4}J_{1\text{-}3}$ = 1.2 Hz, 1H_3), 8.23 (dd, $^{3}J_{13\text{-}14}$ = 8.1 Hz, $^{4}J_{12\text{-}14}$ = 1.9 Hz, 1H_{14}), 7.81 (d, $^{3}J_{9\text{-}10}$ = 8.8 Hz, 1H_9),7.75 (d, $^{3}J_{9\text{-}10}$ = 8.8 Hz, 1H_{10}), 7.74 (dd, $^{3}J_{1\text{-}2}$ = 8.1 Hz, $^{4}J_{1\text{-}3}$ = 1.2 Hz, 1H_1), 7.66 (dd, $^{3}J_{13\text{-}14}$ = 8.1 Hz, $^{3}J_{12\text{-}13}$ = 4.4 Hz, 1H_{13}), 7.46 (dd, $^{3}J_{1\text{-}2}$ = 8.1 Hz, $^{3}J_{2\text{-}3}$ = 6.3 Hz, 1H_2). **MS** (DCI NH_3) $[M+H]^+$ *m/z*= 197.0. **UV-visible** (H_2O) λ_{max} (log *ε*) 213 nm (4.4), 239 nm (4.3), 271 nm (4.4), 308 nm (3.7), 324 nm (3.6), 365 nm (3.3).

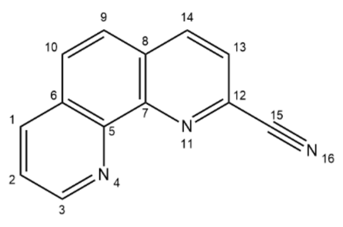

2-cyano-1,10-phenanthroline. To a solution of 10 g (51 mmol) of 1,10-phenanthroline-1-oxide and 10 g (154 mmol) of potassium cyanide dissolved in 80 mL of water were added dropwise 10 mL (86 mmol) of benzoyl chloride under magnetic stirring (total addition required 15 min). A brown solid precipitated. The disappearance of 1,10-phenanthroline-1-oxide and the appearance of 2-cyano-1,10-phenanthroline were followed by TLC with dichloromethane/methanol (98/2 v/v) as eluent. The reaction was stopped when complete disappearance of 1,10-phenanthroline-1-oxide was achieved (ca. 2 h). The precipitate was collected by suction filtration, washed 4 times with water and 5% sodium hydroxide solution alternatively in order to remove the byproducts of benzoyl chloride and dried over vacuum to yield a brown solid (8.3 g, 79% yield). The product was engaged in the next step without further purification. **^{1}H NMR** (400 MHz, $CDCl_3$) δ 9.29 (dd, $^{3}J_{2\text{-}3}$ = 4.4 Hz, $^{4}J_{1\text{-}3}$ = 1.8 Hz, $1H_3$), 8.41 (d, $^{3}J_{13\text{-}14}$ = 8.2 Hz, $1H_{14}$), 8.32 (dd, $^{3}J_{1\text{-}2}$ = 8.1 Hz, $^{4}J_{1\text{-}3}$ = 1.8 Hz, $1H_1$), 7.99 (d, $^{3}J_{9\text{-}10}$ = 7.4 Hz, $1H_{10}$),7.97 (d, $^{3}J_{9\text{-}10}$ = 7.4 Hz, $1H_9$), 7.86 (d, $^{3}J_{13\text{-}14}$ = 8.9 Hz, $1H_{13}$), 7.75 (dd, $^{3}J_{1\text{-}2}$ = 8.1 Hz, $^{3}J_{2\text{-}3}$ = 4.4 Hz, $1H_2$). **^{13}C NMR** (101 MHz, $CDCl_3$) δ 151.47, 137.43, 136.47, 129.99, 126.49, 125.92, 124.33. **MS** (DCI NH_3) $[M+H]^+$ m/z = 206.1. **UV-visible** (CH_2Cl_2) λ_{max} (log ε) 233 nm (4.8), 275 nm (4.7), 316 nm (4.0), 330 nm (3.7), 345 nm (3.3). **IR** 3066-2920*w* (3 peaks, C-H stretching), 2228*w* (C≡N stretching), 1584-1387*m* (4 peaks, C=C arom. stetching), 841s, 778-626*s* (5 peaks, C-H bending).

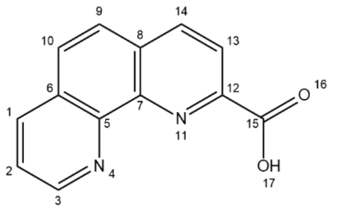

2-carboxy-1,10-phenanthroline. To a solution of 8.3 g (208 mmol) of sodium hydroxide in 50 mL of water was added a solution of 10 g (49 mmol) of 2-cyano-1,10-phenanthroline in 100 mL of 95% ethanol. The mixture was heated to reflux under magnetic stirring. The disappearance of 2-cyano-1,10-phenanthroline was followed by TLC with dichloromethane/methanol (95/5 v/v) as eluent. The reaction was stopped when complete disappearance of 2-cyano-1,10-phenanthroline was achieved (ca. 1.5 h). The ethanol was removed under reduced pressure, and the mixture was acidified with 37% hydrochloric acid to pH≈2 in an ice-water bath. A pale brown solid precipitated, was collected by suction filtration, washed with 10% hydrochloric acid solution and dried over vacuum to yield a pale brown solid (7.7 g, 60% yield). The product was used without further purification. **^{1}H NMR** (400 MHz, DMSO) δ 9.39 (dd, $^{3}J_{2\text{-}3}$ = 5.2 Hz, $^{4}J_{1\text{-}3}$ = 1.5 Hz, $1H_3$), 9.22 (dd, $^{3}J_{1\text{-}2}$ = 8.2 Hz, $^{4}J_{1\text{-}3}$ = 1.5 Hz, $1H_1$), 8.90 (d, $^{3}J_{13\text{-}14}$ = 8.4 Hz, $1H_{14}$), 8.55 (d, $^{3}J_{13\text{-}14}$ = 8.3 Hz, $1H_{13}$), 8.38 (m, $2H_{9\text{-}10}$), 8.31 (dd, $^{3}J_{1\text{-}2}$ = 8.2 Hz, $^{3}J_{2\text{-}3}$ = 5.2 Hz, $1H_2$). **^{13}C NMR** (101 MHz, DMSO) δ 138.70, 130.50, 129.38, 128.32, 127.59, 124.83, 124.04.**MS** (DCI NH_3) $[M+H]^+$ m/z = 225.1. **UV-visible** (H_2O) λ_{max} (log ε) 209 nm (4.3), 228 nm (4.5), 279 nm (4.5), 304 nm (3.9), **IR** 2608*m* (br. O-H stretching), 1742*s* (C=O stretching), 1617-1161*s* (7 peaks, C=C arom. stretching), 875-626*s* (6 peaks, C-H bending).

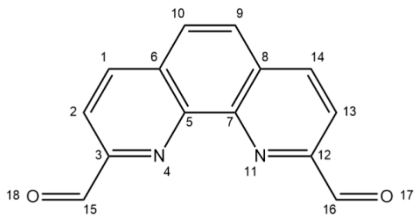

2,9-dicarbaldehyde-1,10-phenanthroline. To a solution of 5.65 g (50.9 mmol) of selenium dioxide in 150 mL dioxane and 10 mL water was added dropwise a solution of 5.0 g (24.0 mmol) 2,9-dimethyl-1,10-phenanthroline in 100 mL dioxane. The mixture was heated to reflux under magnetic stirring. The disappearance of 2,9-dimethyl-1,10-phenanthroline was followed by TLC with dichloromethane/methanol (8/2 v/v) as eluent. The reaction was stopped when complete disappearance of the reactant was achieved (ca. 4 h). The mixture was filtrated on Celite while hot, the filtrate was allowed to cool and a brown solid precipitated. It was collected by filtration, washed with dioxane and dried over vacuum to yield a brown solid (3.90 g, 69% yield). The product was engaged in the next step

without further purification. **^{1}H NMR** (400 MHz, DMSO) δ 10.36 (d, $^4J_{2\text{-}15}$ = 0.9 Hz, 2H$_{15\text{-}16}$), 8.81 (dd, $^3J_{1\text{-}2}$ = 8.3 Hz, $^4J_{2\text{-}15}$ = 0.9 Hz, 2H$_{2\text{-}13}$), 8.33 (d, $^3J_{1\text{-}2}$ = 8.2 Hz,2H$_{1\text{-}14}$), 8.30 (s, 2H$_{9\text{-}10}$). **^{13}C NMR** (101 MHz, DMSO) δ 193.72, 138.45, 129.25, 120.14. **MS** (DCI CH$_4$) [M+H]$^+$ m/z = 237.07.**UV-visible** (DMSO) λ_{max} (log ε) 260 nm (4.4), 287 nm (4.3), 323 nm (4.0), 352 nm (3.5), 399 nm (2.4), 457 nm (2.2). **IR** 1699*s* (C=O stretching), 1594-1083*m* (7 peaks, C=C arom. stretching), 871-726*s* (4 peaks, C-H arom. bending).

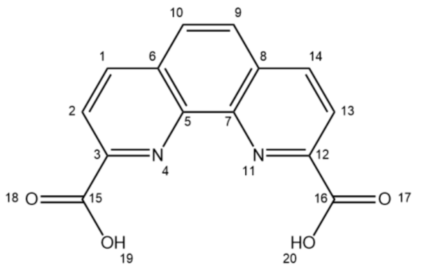

2,9-dicarboxy-1,10-phenanthroline. A solution of 3.90 g of 2,9-dicarbaldehyde-1,10-phenanthroline in 78 mL of nitric acid was heated to reflux. The disappearance of the reactant was followed by NMR, and the reaction was stopped after 4.5 h. The mixture was poured onto ca. 40 mL of ice and allowed to cool: a yellow solid precipitated. It was collected by filtration, washed with 10% hydrochloric acid solution and dried over vacuum to yield a yellow solid (3.80 g, 86% yield). The product was used without further purification. **^{1}H NMR** (300 MHz, DMSO) δ 8.73 (d, $^3J_{1\text{-}2}$ = 8.3 Hz, 2H$_{1\text{-}14}$), 8.41 (d, $^3J_{1\text{-}2}$ = 8.3 Hz, 2H$_{2\text{-}13}$), 8.21 (s, 2H$_{9\text{-}10}$). **^{13}C NMR** (75 MHz, DMSO) δ 166.23, 148.28, 144.72, 138.14, 130.47, 128.39, 123.42. **MS** (DCI CH$_4$) [M+H]$^+$ m/z = 181.08. **UV-visible** (DMSO) λ_{max} (log ε) 282 nm (4.4), 316 nm (3.9), 330 nm (3.7), 347 nm (3.2). **IR** 3445-2436*m* (br. O-H stretching), 1722*s* (C=O stretching), 1409-1036*m* (7 peaks, C=C arom. stretching), 888-700s (4 peaks, C-H bending).

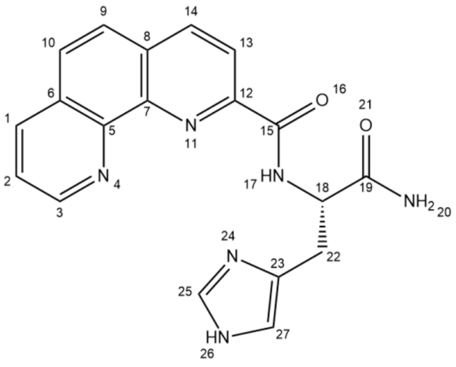

Ligand phenH. In a 20 mL syringe for peptide synthesis, 1 g of MBHA Rink Amide Fmoc-protected resin was deprotected using 5 mL of 20% piperidine in DMF (3x10 min). Deprotected Fmoc was titrated using UV-visible spectroscopy to determine the loading of the resin (0,3 mmol/g). Histidine was coupled to the resin using Fmoc-His(Trt)-OH (4 eq), HBTU (3,9 eq), HOBt (4 eq) and DIPEA (10 eq), in 5 mL of DMF (1 h), and Fmoc-deprotected using 20% piperidine in DMF (3x10 min). Phenanthroline moiety was coupled using 2-carboxy-1,10-phenanthroline (4 eq), PyBOP (4 eq) and DIPEA (10 eq) in 5 mL DMF (overnight). The cleavage from the resin was achieved using 20 mL of TFA:TIPS:H2O (95:2.5:2.5) solution (3 h). The resin was rinced with TFA and the filtrates were collected and concentrated under vacuum. The product was precipitated by addition of ca. 30 mL of cold diethyl ether, collected by centrifugation and dried over vacuum. The product was purified by ChromatoFlash on an INTERCHIM PURIFLASH BIO 100 C18-T (pore size 200 Å, particle size 15 µm, 25 g) using a gradient of solvents A (H2O with 0.1% TFA) and B (MeOH with 0.1% TFA). Fractions containing the product were collected and lyophilized to yield a white solid (72.2 mg, 50% yield). **^{1}H NMR** (400 MHz, D$_2$O) δ 8.92 (d, $^3J_{2\text{-}3}$ = 5.1 Hz, 1H$_3$), 8.67 (d, $^3J_{1\text{-}2}$ = 8.2 Hz, 1H$_1$), 8.64 (s, 1H$_{25}$), 8.25 (d, $^3J_{13\text{-}14}$ = 8.4 Hz, 1H$_{14}$), 8.05 (d, $^3J_{13\text{-}14}$ = 8.3 Hz, 1H$_{13}$), 8.00 (dd, $^3J_{1\text{-}2}$ = 8.0 Hz, $^3J_{2\text{-}3}$ = 5.3 Hz, 1H$_2$), 7.73 – 7.68 (m, 2H$_{9\text{-}10}$), 7.38 (s, 1H$_{27}$), 5.01 (dd, $^3J_{18\text{-}22}$ = 6.9 Hz, $^3J_{18\text{-}22'}$ =8.1 Hz, 1H$_{18}$), 3.53 (dd, $^2J_{22\text{-}22'}$ = 15.3 Hz, $^3J_{18\text{-}22}$ = 6.9 Hz, 1H$_{22}$), 3.41 (dd, $^2J_{22\text{-}22'}$ = 15.4 Hz, $^3J_{18\text{-}22'}$ =8.1 Hz, 1H$_{22'}$). **^{13}C NMR** (101 MHz, D$_2$O) δ 174.10 (C$_{19}$), 164.95 (C$_{15}$), 147.42 (C$_{12}$), 144.76 (C$_3$), 143.60 (C$_1$), 138.48 (C$_{14}$), 138.18 (C$_8$), 137.88 (C$_6$), 133.56 (C$_{25}$), 130.33 (C$_7$), 129.24 (C$_5$), 128.53 (C$_{23}$), 128.04 (C$_9$), 127.07 (C$_{10}$), 124.71 (C$_2$), 122.29 (C$_{13}$), 117.22 (C$_{27}$), 52.80 (C$_{18}$), 26.71 (C$_{22}$). **MS** (ESI) [M+H]$^+$ m/z = 361.1. **UV-visible** (H$_2$O) λ_{max} (log ε) 208 nm (4.5), 231 nm (4.7), 280 nm (4.5), 313 nm (3.9), 328 nm (3.6), 343 nm (3.1). IR 3283-2627*w* (br. N-H stretching), 1662*s* (C=O stretching), 1534-

1416*m* (3 peaks, C=C arom. stretching), 1181-1126*s* (2 peaks, C-F stretching), 867-626*m* (5 peaks, C-H bending).

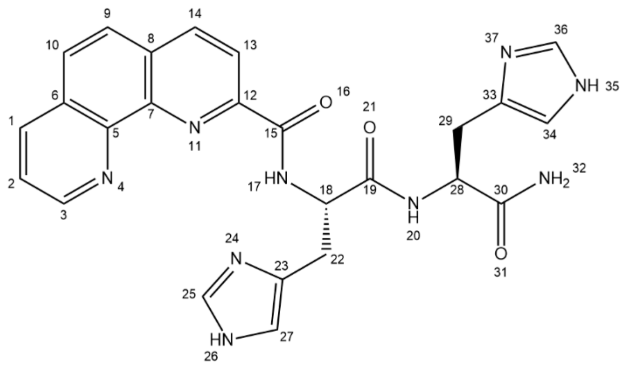

Ligand phenHH. Ligand phenHH was synthesized and purified in a similar way as phenH, by doubling the histidine coupling step. A white solid was obtained (46%). **^{1}H NMR** (400 MHz, D_2O) δ 9.24 (d, $^3J_{2\text{-}3}$ = 5.3 Hz, 1H$_3$), 9.21 (d, $^3J_{1\text{-}2}$ = 8.2 Hz, 1H$_1$), 8.75 (d, $^3J_{13\text{-}14}$ = 8.5 Hz, 1H$_{14}$), 8.64 (d, $^4J_{25\text{-}27}$ = 1.4 Hz, 1H$_{25}$), 8.52 (d, $^4J_{34\text{-}36}$ = 1.4 Hz, 1H$_{36}$), 8.47 (d, $^3J_{13\text{-}14}$ = 8.4 Hz, 1H$_{13}$), 8.33 (dd, $^3J_{1\text{-}2}$ = 8.3 Hz, $^3J_{2\text{-}3}$ = 5.4 Hz, 1H$_2$), 8.28 – 8.18 (m, 2H$_{9\text{-}10}$), 7.35 (d, $^4J_{25\text{-}27}$ = 1.4 Hz, 1H$_{27}$), 7.30 (d, $^4J_{34\text{-}36}$ = 1.4 Hz, 1H$_{34}$), 5.02 (t, $^3J_{18\text{-}22}$ = 7.5 Hz, 1H$_{18}$), 4.78 (in D_2O peak, 1H$_{28}$), 3.50 (d, $^3J_{18\text{-}22}$ = 7.7 Hz, 2H$_{22}$), 3.31 (dd, $^2J_{29\text{-}29'}$ = 15.5, $^3J_{28\text{-}29}$ = 5.9 Hz, 1H$_{29}$), 3.19 (dd, $^2J_{29\text{-}29'}$ = 15.5, $^3J_{28\text{-}29'}$ = 8.8 Hz, 1H$_{29'}$). **^{13}C NMR** (75 MHz, D_2O) δ 173.64 (C$_{30}$), 171.67 (C$_{19}$), 165.25 (C$_{15}$), 148.16 (C$_{12}$), 146.41 (C$_1$), 143.27 (C$_3$), 139.35 (C$_{14}$), 137.77 (C$_8$), 137.05 (C$_6$), 133.62 (C$_{25}$), 133.37 (C$_{36}$), 131.40 (C$_7$), 130.24 (C$_5$), 129.28 (C$_9$), 128.37 (C$_{23}$), 128.27 (C$_{33}$), 127.14 (C$_{10}$), 125.06 (C$_2$), 123.23 (C$_{13}$), 117.25 (C$_{27}$), 117.22 (C$_{34}$), 53.12 (C$_{18}$), 52.19 (C$_{28}$), 26.46 (C$_{29}$), 26.22 (C$_{22}$). **MS** (ESI) $[M+H]^+$ *m*/*z*= 498.2.**UV-visible** (H_2O) λ_{max} (log ε) 210 nm (4.6), 230 nm (4.6), 279 nm (4.5), 314 nm (3.8), 328 nm (3.6), 343 nm (3.1). **IR** 3272-2621*w* (6 peaks, br. N-H stretching), 1663*s* (C=O stretching),1655*s* (C=O stretching), 1535-1418*m* (3 peaks, C=C arom. stretching), 1185-1128*s* (2 peaks, C-F stretching), 870-626*m* (5 peaks, C-H bending).

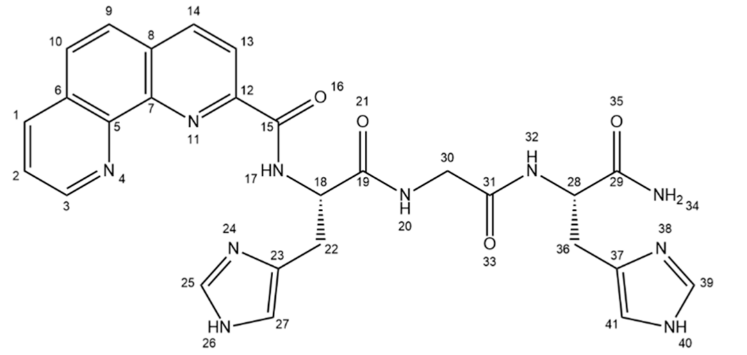

Ligand phenHGH. Ligand phenHGH was synthesized and purified in a similar way as phenHH, by adding a glycine coupling step between the 2 histidine coupling steps: glycine was coupled to the resin using Fmoc-Gly-OH (4 eq), HBTU (3,9 eq), HOBt (4 eq) and DIPEA (10 eq), in 5 mL of DMF (1 h), and Fmoc-deprotected using 20% piperidine in DMF (3x10 min). A white solid was obtained (106 mg, 50% yield). **^{1}H NMR** (400 MHz, D_2O) δ 9.22 (d, $^3J_{2\text{-}3}$ = 5.4 Hz, 1H$_3$), 9.15 (d, $^3J_{1\text{-}2}$ = 8.3 Hz, 1H$_1$), 8.69 (d, $^3J_{13\text{-}14}$ = 8.4 Hz, 1H$_{14}$), 8.63 (s, 1H$_{25}$), 8.57 (s, 1H$_{39}$), 8.42 (d, $^3J_{13\text{-}14}$ = 9.2 Hz, 1H$_{13}$), 8.30 (dd, $^3J_{1\text{-}2}$ = 8.3 Hz, $^3J_{2\text{-}3}$ = 5.4 Hz, 1H$_2$), 8.18 (s, 2H$_{9\text{-}10}$), 7.36 (s, 1H$_{27}$), 7.29 (s, 1H$_{41}$), 5.10 (dd, $^3J_{18\text{-}22'}$ = 8.7 Hz, $^3J_{18\text{-}22}$ = 6.2 Hz, 1H$_{18}$), 4.72 (dd, $^3J_{28\text{-}36'}$ = 8.5 Hz, $^3J_{28\text{-}36}$ = 5.7 Hz, 1H$_{28}$), 4.08 (d, $^2J_{30\text{-}30'}$ = 17.0 Hz, 1H$_{30}$), 3.98 (d, $^2J_{30\text{-}30'}$ = 17.0 Hz, 1H$_{30'}$), 3.60 (dd, $^2J_{22\text{-}22'}$ = 15.4, $^3J_{18\text{-}22}$ = 6.2 Hz, 1H$_{22}$), 3.48 (dd, $^2J_{22\text{-}22'}$ = 15.5 Hz, $^3J_{18\text{-}22'}$ = 8.7 Hz, 1H$_{22'}$), 3.32 (dd, $^2J_{36\text{-}36'}$ = 15.5 Hz, $^3J_{28\text{-}36}$ = 5.7 Hz, 1H$_{36}$), 3.20 (dd, $^2J_{36\text{-}36'}$ = 15.6 Hz, $^3J_{28\text{-}36'}$ = 8.5 Hz, 1H$_{36'}$). **^{13}C NMR** (101 MHz, D_2O) δ 174.13 (C$_{29}$), 172.43 (C$_{19}$), 171.06 (C$_{31}$), 165.56 (C$_{15}$), 148.04 (C$_{12}$), 146.00 (C$_1$), 143.55 (C$_3$), 139.22 (C$_{14}$), 137.88 (C$_8$), 137.24 (C$_6$), 133.55 (C$_{25}$), 133.37 (C$_{39}$), 131.26 (C$_7$), 130.10 (C$_5$), 129.09 (C$_9$), 128.65 (C$_{23}$), 128.42 (C$_{37}$), 127.17 (C$_{10}$), 125.02 (C$_2$), 123.13 (C$_{13}$), 117.24 (C$_{27}$), 117.11 (C$_{41}$), 53.10 (C$_{18}$), 52.26 (C$_{28}$), 42.38 (C$_{30}$), 26.35 (C$_{22}$), 26.31 (C$_{36}$). **MS** (ESI) $[M+H]^+$ *m*/*z*= 555.2. **UV-visible** (H_2O) λ_{max} (log ε) 205 nm (4.6), 231 nm (4.7), 281 nm (4.5), 313 nm (3.9), 328 nm (3.6), 344 nm (3.1). **IR** 3277-

2621*w* (6 peaks, br. N-H stretching), 1662*s* (C=O stretching),1628*s* (C=O stretching), 1537-1415*m* (3 peaks, C=C arom. stretching), 1183-1125*s* (2 peaks, C-F stretching), 870-626*m* (5 peaks, C-H bending).

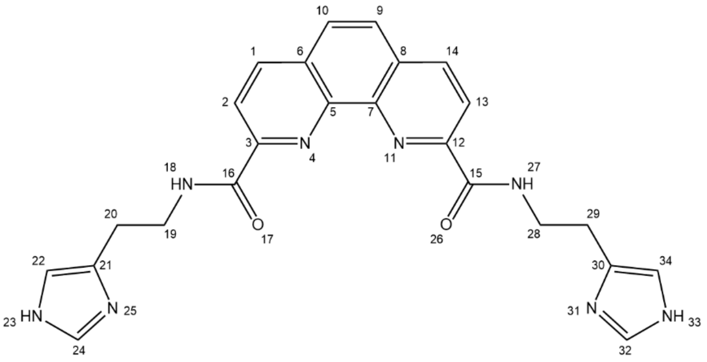

Ligand H′phenH′. A solution of 0.24 g (0.89 mmol) of 2,9-dicarboxy-1,10-phenanthroline in 5 mL of anhydrous DMF was heated at 40°C. 0.61 g (3.8 mmol) of 1,1′-carbonyldiimidazole were added by portions and the mixture was stirred at 40°C for 1.5 h. 0.42 g (2.3 mmol) of histamine dihydrochloride were added and the mixture was stirred at 40°C overnight. 4 mL of water were added, and the solvents were removed under reduced pressure. A solid precipitated upon addition of 5 mL of 1 M sodium carbonate solution: it was collected by suction filtration, washed with 1 M sodium carbonate and dried over vacuum to yield a pale brown powder (0.23 g, 57% yield). The product was used without further purification. **^{1}H NMR** (400 MHz, DMSO) δ 9.58 (s, $2H_{18\text{-}27}$), 8.73 (d, $^3J_{1\text{-}2}$ = 8.1 Hz, $2H_{1\text{-}14}$), 8.45 (d, $^3J_{1\text{-}2}$ = 8.2 Hz, $2H_{2\text{-}13}$), 8.18 (s, $2H_{9\text{-}10}$), 7.57 (s, $2H_{24\text{-}32}$), 6.93 (s, $2H_{22\text{-}34}$), 3.68 (m, $4H_{19\text{-}28}$), 2.92 (t, J = 7.4 Hz, $4H_{20\text{-}29}$).**^{13}C NMR** (101 MHz, DMSO) δ 163.76 ($C_{15\text{-}16}$), 149.62 ($C_{3\text{-}12}$), 149.60 ($C_{22\text{-}34}$), 143.66 ($C_{6\text{-}8}$), 138.15 ($C_{1\text{-}14}$), 134.80 ($C_{24\text{-}32}$), 130.17 ($C_{5\text{-}7}$), 127.82 ($C_{9\text{-}10}$), 120.91 ($C_{2\text{-}13}$), 40.14 ($C_{19\text{-}28}$), 26.95 ($C_{20\text{-}29}$). **MS** (ESI) $[M+H]^+$ *m/z* = 455.2. **UV-visible** (MeOH) λ_{max} (log ε) 211 nm (4.6), 238 nm (4.7), 248 nm (4.7), 281 nm (4.5), 316 nm (4.1), 323 nm (4.0), 330 nm (3.8), 346 nm (3.4). **IR**3299-2890*w* (6 peaks, br. N-H stretching), 1650*s* (C=O stretching),1537-1436*m* (5 peaks, C=C arom. stretching), 867-622*m* (4 peaks, C-H bending).

2. Supplementary schemes, figures and tables

Ligands phenH, phenHH and phenHGH synthesis

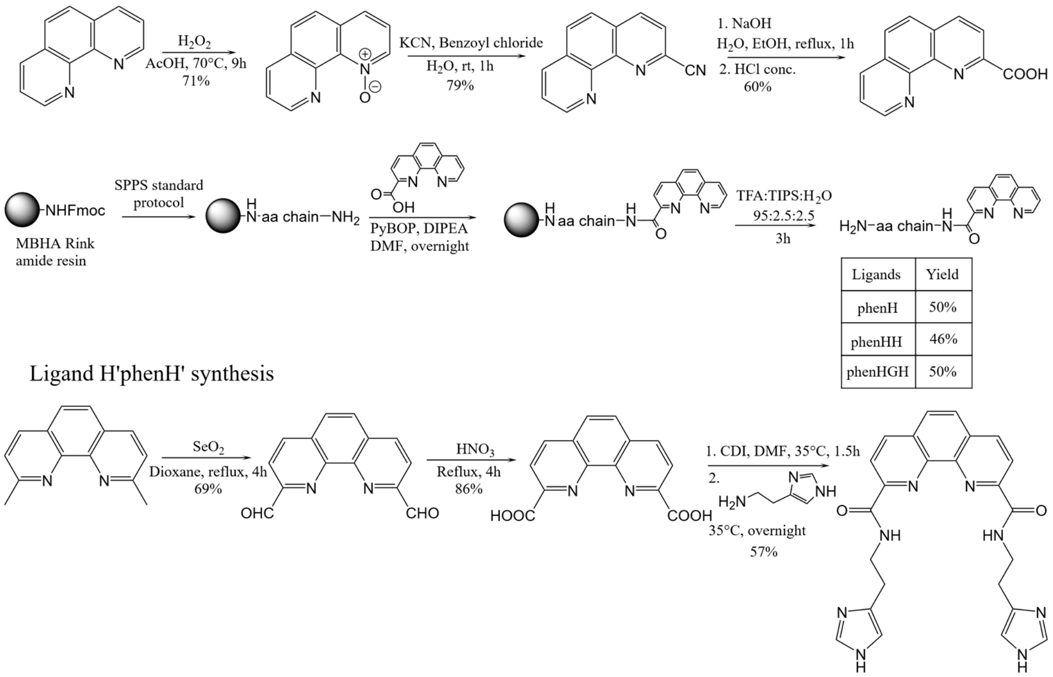

Ligands	Yield
phenH	50%
phenHH	46%
phenHGH	50%

Scheme S1. Synthesis of 2-carboxy-1,10-phenanthroline, ligands phenH, phenHH and phenHGH, 2-9-dicarboxy-1,10-phenanthroline, ligand H'phenH'.

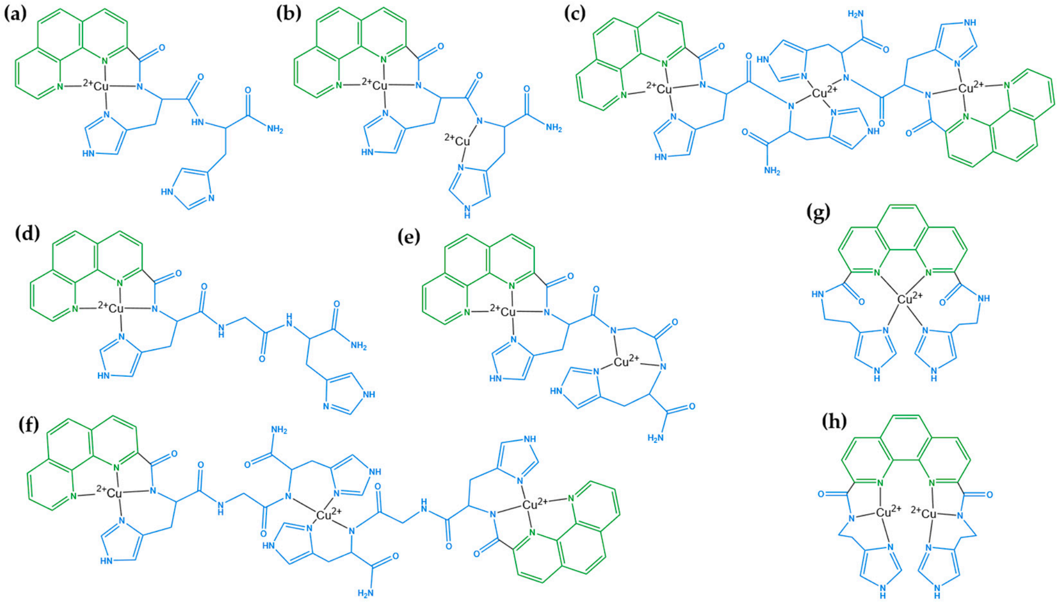

Scheme S2. Proposed structures of (**a**) Cu(II)(phenHH), (**b**) $Cu(II)_2$(phenHH), (**c**) $Cu(II)_3(phenHH)_2$, (**d**) Cu(II)(phenHGH), (**e**) $Cu(II)_2$(phenHGH), (**f**) $Cu(II)_3(phenHGH)_2$, (**g**) Cu(II)(H'phenH'), (**h**) $Cu(II)_2$(H'phenH').

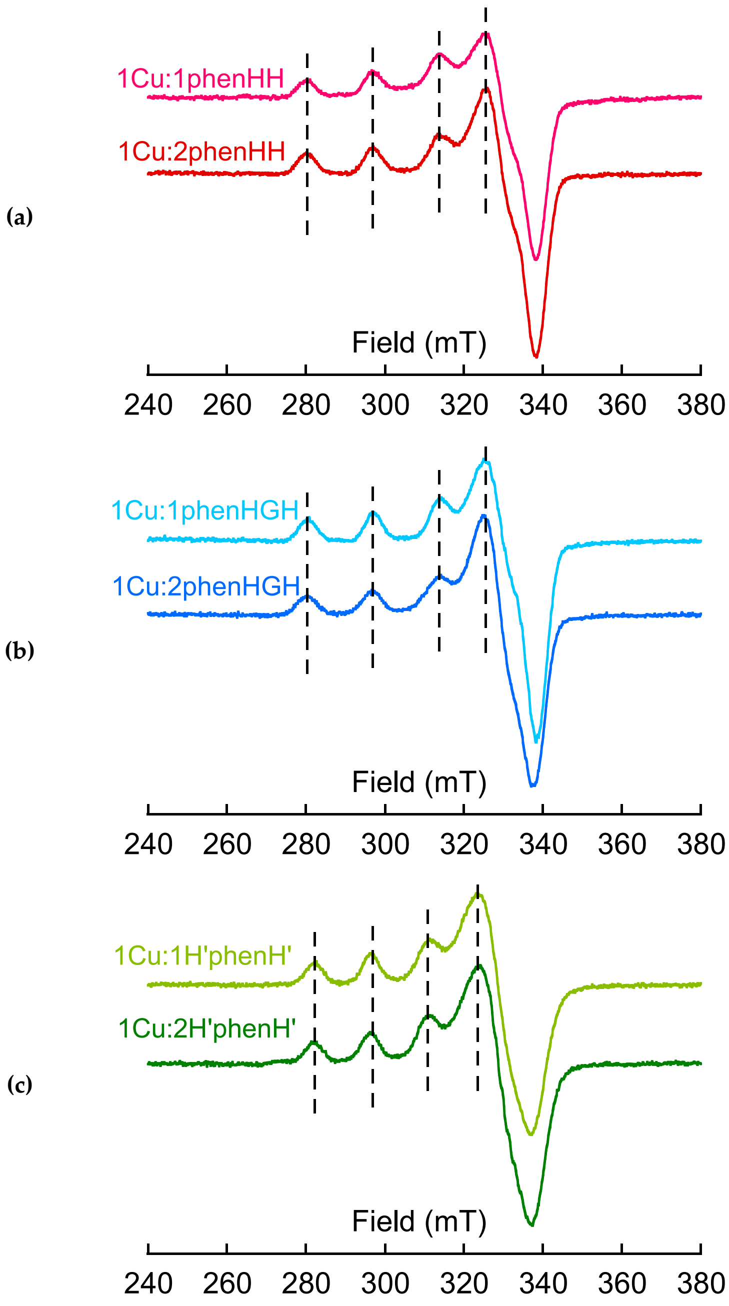

Figure S1. 9 GHz-band EPR spectra of Cu(II) with 1 or 2 equivalents of the ligands ((a) phenHH, (b) phenHGH, (c) H′phenH′). [Cu] = 500 µM (Cu isotopes in natural abundance), [ligand] = 500 or 1000 µM, [HEPES] = 50 mM, pH 7.4, 10% of glycerol as cryoprotectant. Attenuation = 16 dB and amplitude modulation = 5 G.

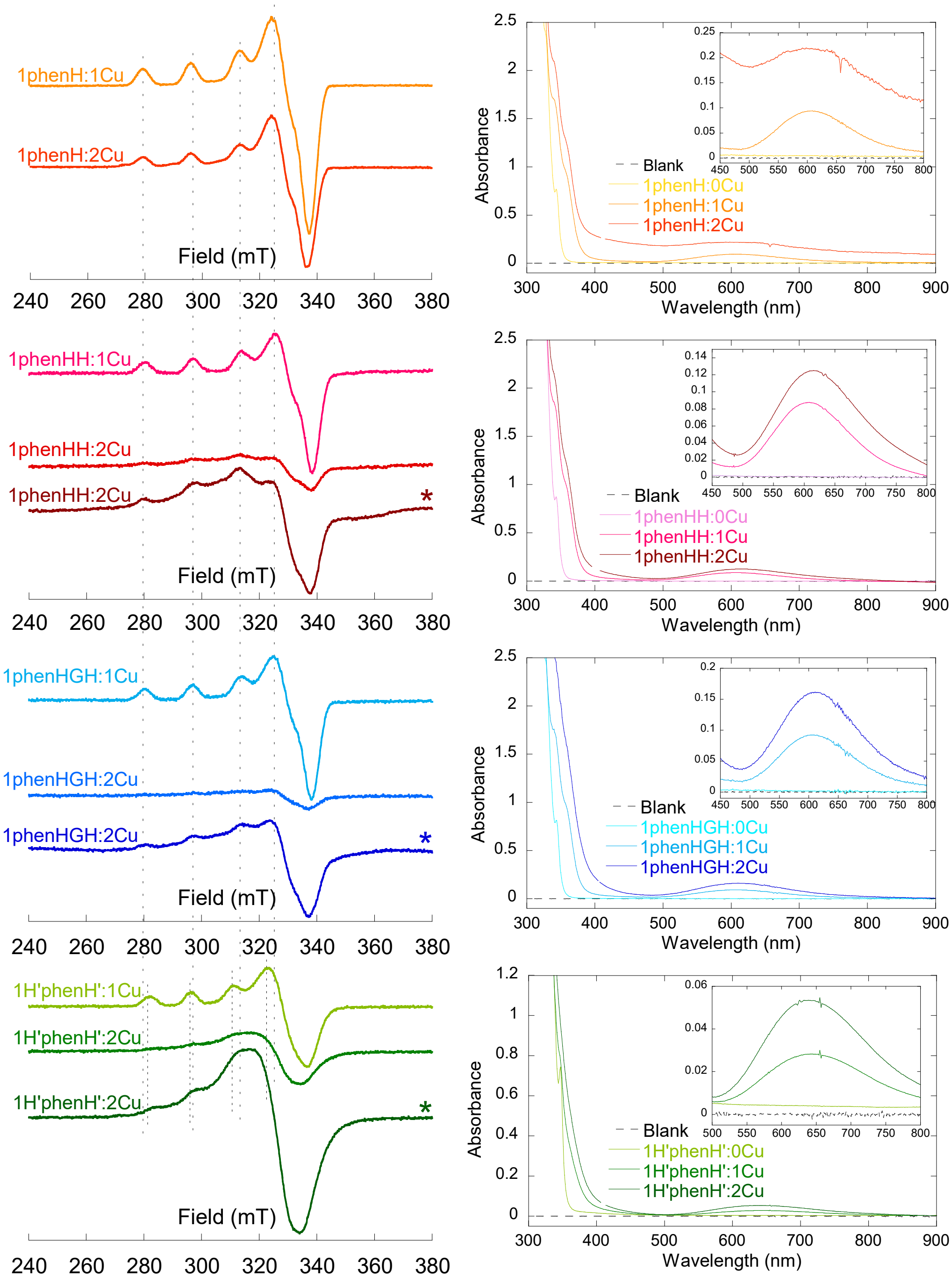

(**a**) (**b**)

Figure S2. (**a**) 9 GHz-band EPR spectra of the ligands with 1 or 2 equivalents of Cu(II). [Cu] = 500 µM (Cu isotopes in natural abundance), [ligand] = 250 or 500 µM, [HEPES] = 50 mM, pH 7.4, 10% of glycerol as cryoprotectant. Attenuation = 16 dB and amplitude modulation = 5 G except for the spectra marked with (*): attenuation = 10 dB and amplitude modulation = 10 G. (**b**) UV-visible spectra of the ligands with 0, 1 or 2 equivalents of Cu(II). [phenH] = [phenHH] = [phenHGH] = 0.8 mM, [H'phenH'] = 0.25 mM, [HEPES] = 100 mM, pH 7.1.

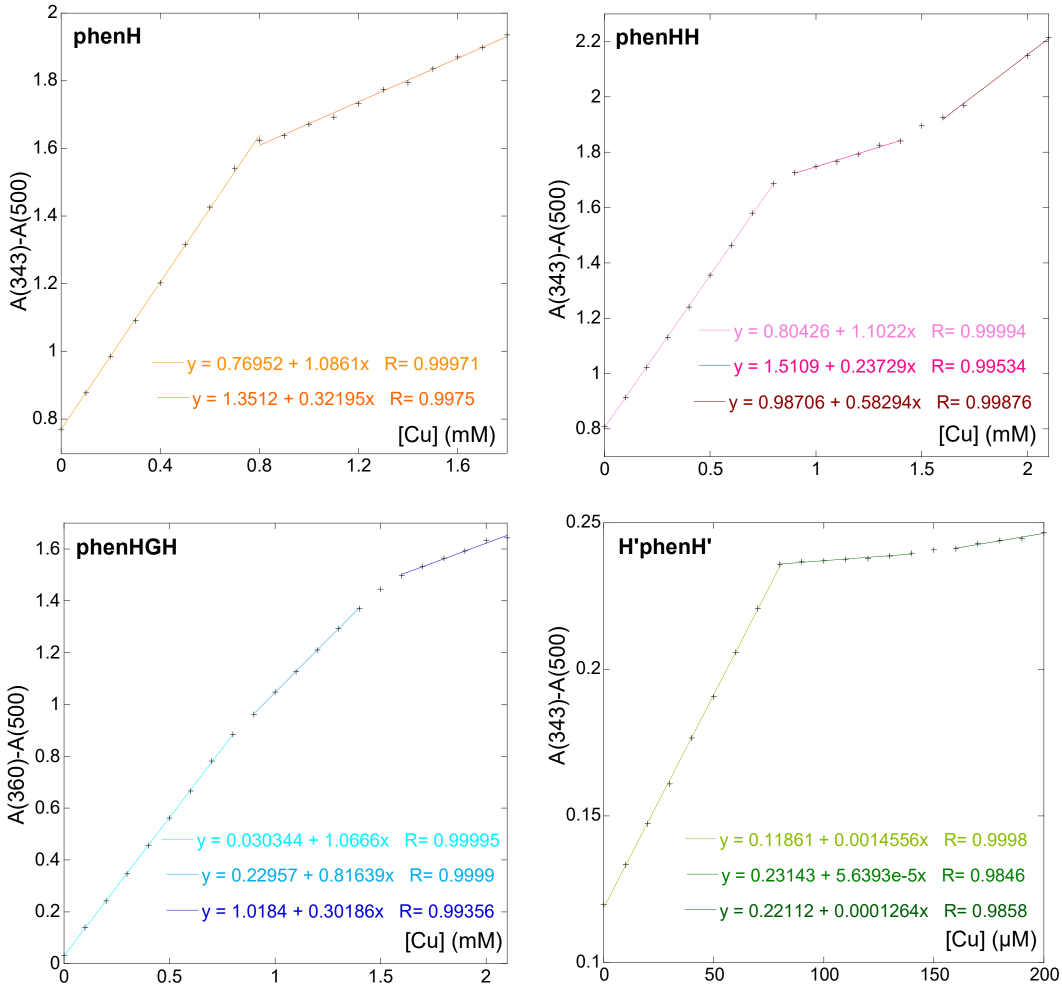

Figure S3. Titration curves of the ligands by Cu(II), monitored by UV-visible spectroscopy at 343 nm for phenH, phenHH and H′phenH′ and 360 nm for phenHGH, with single reference wavelength correction at 500 nm. Ligand concentration was approximatively 0.8 mM for phenH, phenHH and phenHGH, and 80 µM for H′phenH′ (with 1% methanol). [HEPES] = 100 mM, pH 7.1. Colored lines represent the linear fits of the data.

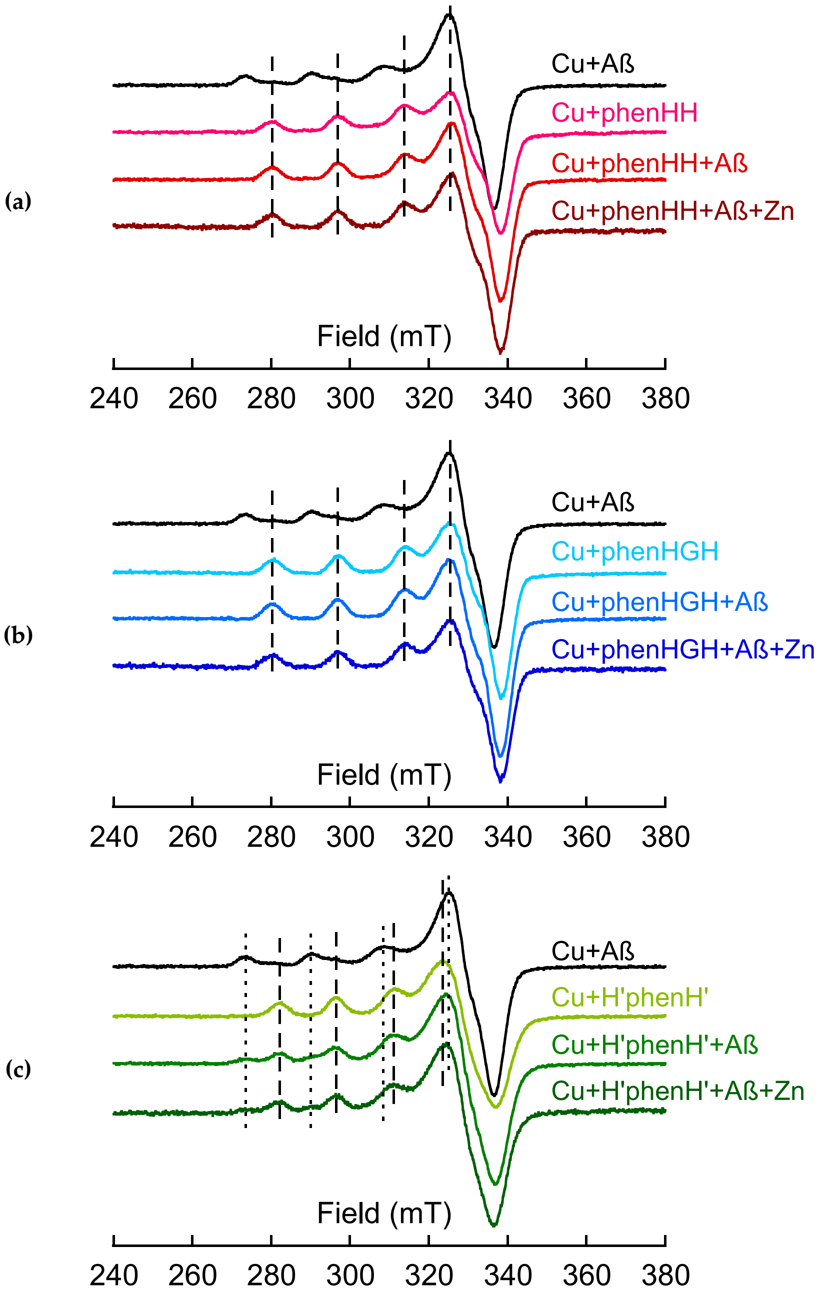

Figure S4. 9 GHz-band EPR spectra of Cu(II) with 1 equivalent of the ligands ((a) phenHH, (b) phenHGH, (c) H'phenH'), in presence of Aβ and Aβ+Zn(II). [Cu] = [ligand] = [Aβ] 500 μM (Cu isotopes in natural abundance), except for the experiments with Aβ+Zn(II) : [Cu] = [ligand] = [Aβ] = [Zn] = 250 μM. [HEPES] = 50 mM, pH 7.4, 10% of glycerol as cryoprotectant. Attenuation = 16 dB and amplitude modulation = 5 G.

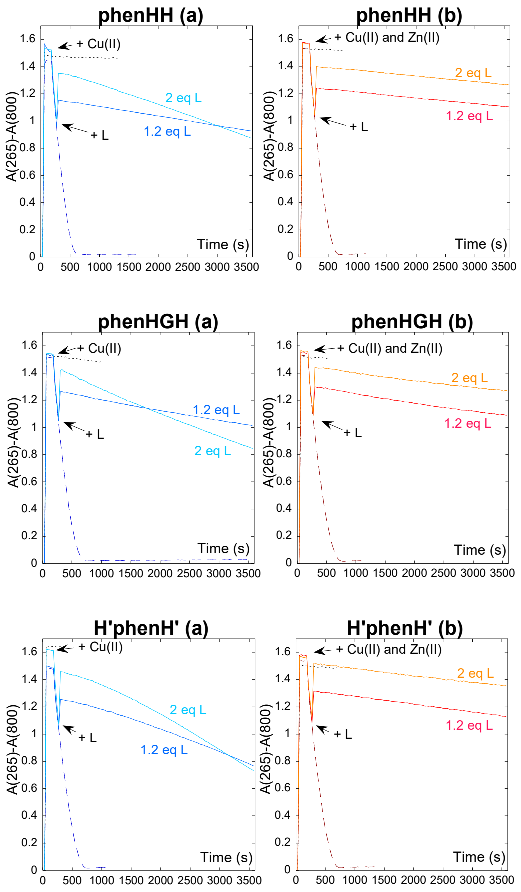

Figure S5. Kinetics of ascorbate consumption, starting from a mixture of Cu(II) and Cu(I) without (**a**) or with (**b**) Zn(II), without (dashed line) or with (plain line) 1.2 or 2 equivalents of L (ligand), followed by UV-vis spectroscopy at 265 nm with a background correction at 800 nm. $[AscH^-]$ = 100 µM, [Cu] = 10 µM, [Aβ] = [Zn] = 12 µM, [L] = 12 or 20 µM, [HEPES] = 100 mM, pH 7.1. Black dotted lines indicate the control experiment with ascorbate only.

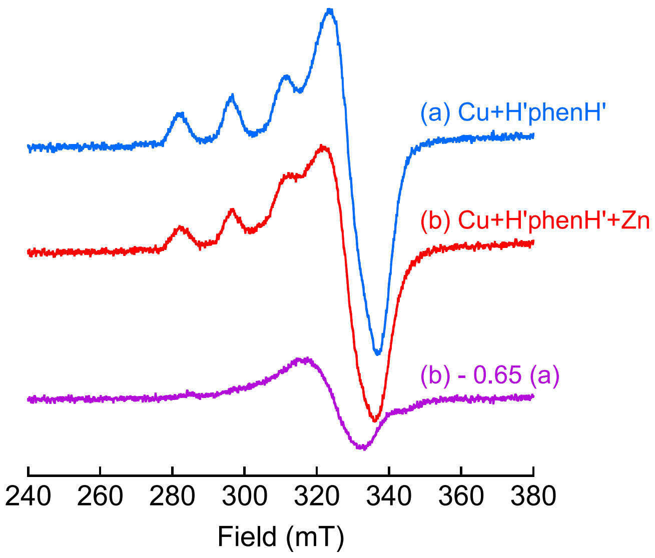

Figure S6. 9 GHz-band EPR spectra of Cu(II) with 1 equivalent of H'phenH') in presence of Zn(II). In purple: substraction of 0.65 (a) spectrum to (b) spectrum. [Cu] = [ligand] = [Zn] = 500 µM (Cu isotopes in natural abundance). [HEPES] = 50 mM, pH 7.4, 10% of glycerol as cryoprotectant. Attenuation = 16 dB and amplitude modulation = 5 G.

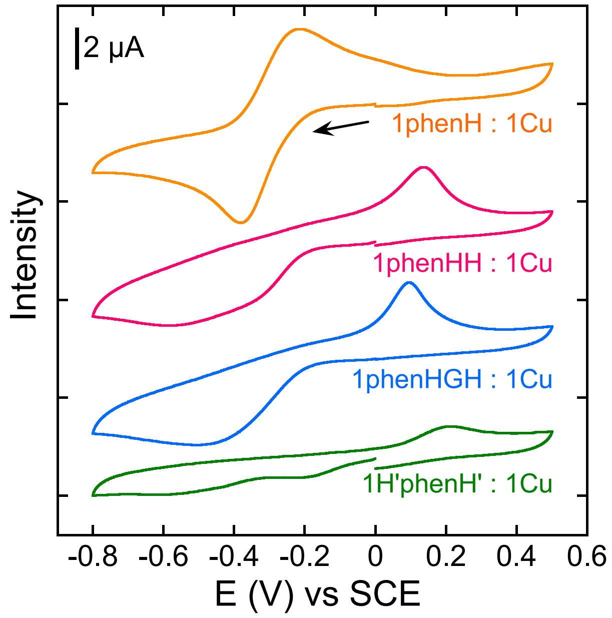

Figure S7. Cyclic voltammograms of Cu in presence of 1 equivalent of phenH, phenHH, phenHGH and H′phenH′. Scan rate: 100 mV/s. [Cu] = 0.5 mM, [L] = 0.5 mM, [HEPES] = 50 mM, pH 7.1, except for H′phenH′ : [Cu] = 0.1 mM, [L] = 0.1 mM, [HEPES] = 50 mM, pH 7.1 + 1% methanol.

Table S1. UV-visible (ε are calculated by dividing absorbance at $\lambda_{max}^{d\text{-}d}$ by Cu(II) concentration but are not representative of a certain complex, since more than one complex may be in solution) and EPR parameters of the ligands in presence of 1 or 2 equivalents of Cu(II).

Ligands	Eq Cu(II)	UV-vis parameters	EPR parameters		
		$\lambda_{max}^{d\text{-}d}$ (nm) (ε (M^{-1} cm^{-1}))	$g_{//}$	$A_{//}$ (10^{-4} cm^{-1})	$g_{\perp}$
phenH	1	608 (117)	2.21	178	2.05
	2	605 (136)	2.22	178	2.06
phenHH	1	607 (109)	2.22	176	2.05
	2	615 (78)	2.22	178	2.06.
phenHGH	1	605 (115)	2.22	176	2.05
	2	612 (100)	2.22	174	2.06
H′phenH′	1	642 (117)	2.23	151	2.06
	2	640 (107)	2.21	155	2.08
